# Surgeon General of the U. S.

**Published:** 1882-08-20

**Authors:** 


### *SURGEON GENERAL OF THE UNITED STATES.*

Sugeon General Barnes being sixty-four years old has been placed on the retired list. He has served as Surgeon General for twenty-two years.

The officer appointed to fill his place is Dr. Charles H. Crane, of New York, who has been acting as Assistant Surgeon General since the close of the late war.

